# Environmental Isolation of *Cryptococcus gattii* VGII from Indoor Dust from Typical Wooden Houses in the Deep Amazonas of the Rio Negro Basin

**DOI:** 10.1371/journal.pone.0115866

**Published:** 2015-02-17

**Authors:** Fábio Brito-Santos, Gláucia Gonçalves Barbosa, Luciana Trilles, Marília Martins Nishikawa, Bodo Wanke, Wieland Meyer, Filipe Anibal Carvalho-Costa, Márcia dos Santos Lazéra

**Affiliations:** 1 Mycology Laboratory, Evandro Chagas Clinical Research Institute, FIOCRUZ, Rio de Janeiro, Brazil; 2 Molecular Mycology Research Laboratory, Centre for Infectious Diseases and Microbiology, Sydney Medical School-Westmead Hospital, Marie Bashir Institute for Infectious Diseases and Biosecurity, The University of Sydney, Westmead Millennium Institute, Sydney, New South Wales, Australia; 3 Laboratory of Molecular Epidemiology and Systematics, Institute Oswaldo Cruz, FIOCRUZ, Rio de Janeiro, Brazil; 4 National Institute for Quality Control in Health, INCQS/FIOCRUZ, Rio de Janeiro, Brazil; University of Minnesota, UNITED STATES

## Abstract

Cryptococcosis is a human fungal infection of significant mortality and morbidity, especially in the meningoencephalitis form. Cryptococcosis is distributed worldwide and its agents, *C. neoformans* and *C. gattii*, present eight major molecular types—VNI-VNIV and VGI-VGIV respectively. The primary cryptococcosis caused by molecular type VGII (serotype B, MAT alpha) prevails in immunocompetent patients in the North and Northeast of Brazil, revealing an endemic regional pattern to this molecular type. Since 1999, *C. gattii* VGII has been involved in an ongoing outbreak in Canada, and is expanding to the Northwest of the United States, two temperate regions. Exposure to propagules dispersed in the environment, related to various organic substrates, mainly decomposing wood in and around dwellings, initiates the infection process. The present study investigated the presence of the agents of cryptococcosis in dust from dwellings in the upper Rio Negro, municipality of Santa Isabel do Rio Negro in Amazonas state. Indoor dust was collected from 51 houses, diluted and plated on bird seed agar. Dark brown colonies were identified phenotypically, and genotypically by *URA5* restriction fragment length polymorphism analysis and multilocus sequence typing (MLST). The mating type was identified using pheromone-specific primers. Three of the 51 houses were positive for *C. gattii* molecular type VGII, *MATα* and *MAT**a***, showing a high prevalence of this agent. MLST studies identified eight subtypes, VGIIb (ST7), VGIIa (ST20), (ST5) and 5 new subtypes unique to the region. For the first time in the state of Amazonas, *C. gattii* VGII *MATα* and *MAT**a*** were isolated from the environment and correlates with endemic cryptococcosis in this state. This is the first description of MLST subtypes on environmental isolates in the Brazilian Amazon, indicating domiciliary dust as a potential source for human infection with different subtypes of *C. gattii* VGII *MATα* and *MAT**a***.

## Introduction

Cryptococcosis is a life-threatening systemic mycosis affecting humans and animals worldwide. The disease is acquired by inhalation of infectious propagules (desiccated yeasts cells or basidiospores) of the members of the *Cryptococcus neoformans*/*C*. *gattii* species complex from the environment. Its most frequent clinical manifestation is meningoencephalitis [1]. Cryptococcosis by *C*. *neoformans* is cosmopolitan, affecting mainly immunocompromised individuals, especially patients with AIDS [2]. On the other hand, *C*. *gattii* causes predominantly a primary infection in immunocompetent individuals, previously associated with tropical and subtropical climates, but now gaining prominence as an important cause of human and veterinary disease in temperate regions of North America [3–7].

Molecular epidemiological studies have identified eight major molecular types within the *Cryptococcus neoformans/C*. *gattii* species complex. *C*. *neoformans* is classified into the major molecular types VNI/AFLP1 and VNII/AFLP1A and AFLP1B (serotype A), VNIII/AFLP3 (serotype AD), and VNIV/AFLP2 (serotype D), while *C*. *gattii* correspond to VGI/AFLP4, VGII/AFLP6, VGIII/AFLP5, and VGIV/AFLP7, all corresponding to serotypes B and/or C [8]. In addition there are inter-specific serotype BD or AB hybrids, corresponding to AFLP8 and AFLP9, respectively [9,10]. To globally standardize genotyping of the *C*. *neoformans/C*. *gattii* species complex, a MLST scheme was established by the ISHAM working group “Genotyping *C*. *neoformans* and *C*. *gattii*” based on variable regions of seven independent genetic loci: *CAP59*, *GPD1*, *LAC1*, *PLB1*, *SOD1*, *URA5* and the IGS1 region, taking advantage of the high discriminatory power and good reproducibility between different laboratories [8]. The allele (AT) and sequence types (ST) of the ISHAM consensus MLST scheme can be determined via the web page at http://mlst.mycologylab.org/.

Cryptococcosis by *C*. *gattii* in Brazil is endemic and shows a regional pattern, being more common in the North (N) and Northeast (NE) of the country [11]. One hundred thirty four (134) cases have been diagnosed in the Amazon region, including the states of Pará and Amazonas [12–15]. In these states, as well as in other states of the N and NE regions (Roraima, Maranhão and Piauí), noteworthy was the emergence of cryptococcosis in immunocompetent (HIV-negative) children in about one fifth of the reported cases [12, 15–16], suggesting that natural infection occurs early in life. The dynamics of natural infections by *C*. *gattii* in these regions is not well known and requires more specific environmental studies to detect possible outbreaks. Indeed, environmental sources related to trees colonized by the agents of cryptococcosis have been described in Roraima [17], Pará [18] and Piauí [19] states.

Pioneering studies of cryptococcosis in AIDS in Central Africa and Brazil demonstrated the risk of those patients to acquire cryptococcosis from indoor dust [20, 21]. The current study investigated the presence of *C*. *neoformans* and *C*. *gattii* in dwellings of the hinterland of the Brazilian Amazon, and characterized the molecular subtypes and mating types of the obtained isolates.

## Methods

### Studied Region

**Fig 1:**
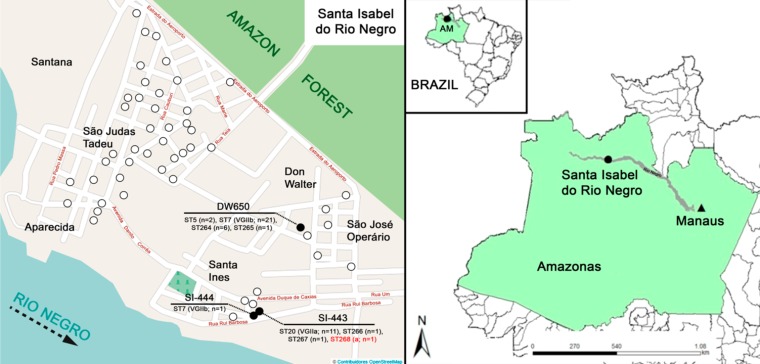
Sampling locations in the city of Santa Isabel do Rio Negro (circles) with the 3 positive houses (dark circles), DW650, SI-444 and SI-443 with the respective sequence types (ST) identified amongst the samples, and location of Santa Isabel do Rio Negro in the Amazonas state, Northern Brazil. The maps were adapted from Bing Maps to reflect the specific study site situation.

The study was conducted in the city of Santa Isabel do Rio Negro, Amazonas state, located 620 km straight or 772 km by waterway away from Manaus, the capital of the Amazonas state, and about 1,000 km far from the Atlantic Ocean coast of the Brazilian Northern region (Fig. 1). The municipality of this city has the following characteristics: land area of 63,127 km², tropical rainforest climate, rainy and humid; maximum temperature 32.6°C, minimum 21.5°C; altitude: 21m above sea level; Cartesian coordinates: 0° 28′ south latitude and 65° 32′ longitude west of Greenwich. The city settlement occurred approximately one hundred years ago and the population reaches 19,292 inhabitants [22], with the majority of them living in wooden houses. The research was conducted in an urban area (coordinates 65W 01’ 08”- 0S 24’ 51”), so special permission from authorities was not required. However, the study had the approval of the Municipal Health Department of the city, as well as prior ethical approval in the context of the project “Study of the health conditions of the municipality of Santa Isabel do Rio Negro” (Oswaldo Cruz Foundation Ethical Research Committee, Rio de Janeiro, Brazil, reference n° CAAE 0011.0.009.000–03).

### Sampling and Cryptococcal isolation

Dust samples were collected from 51 houses randomly chosen in different neighborhoods of the city of Santa Isabel do Rio Negro, to investigate the presence of the agents of cryptococcosis, *C*. *neoformans* and *C*. *gattii*. One sample per house was obtained combining dust from all rooms swept with a different broom per house to prevent cross contamination between houses, and the material was stored in sterile plastic bags (Nasco/WHIRL-PARK). The location of the selected households was obtained using a GPS (Garmin Etrex H). Cryptococcal cells were isolated as described previously [21, 23]. Briefly, 1g of each dust sample was suspended in 50 ml NaCl 0.9% with 0.2 g of chloramphenicol, followed by manual shaking for 5 minutes. After resting 30 minutes, 1 ml of the supernatant was plated onto 10 Niger Seed Agar (NSA) plates (0.1 ml each) [24]. The plates were then incubated at 25°C and checked daily for brown colonies for 5 days. Each phenol oxidase-positive/brown colony was sub-cultured individually on NSA medium for phenotypical and molecular characterization. The isolates were preserved in skim milk at -20°C and in glycerol at -70°C and deposited in the Culture Collection of Pathogenic Fungi (CFP) of the Mycology Laboratory, IPEC/FIOCRUZ. The limit for the detection of phenol oxidase-positive colonies was 50 CFU per gram of sample.

### Phenotypic Identification

Brown colonies were purified and tested for urease production on Christensen urea agar [25], and for carbon and nitrogen compound assimilation using VITEK 2-BioMerieux System (VITEK 2, ICB, bioMerieux, Durham, USA). The species *C*. *neoformans* and *C*. *gattii* were differentiated on canavanine-glycine-bromothymol blue (CGB) medium [26].

### Identification of the Major Molecular Types

After DNA extraction [27], the major molecular types of all studied isolates were identified by *URA5*-RFLP analysis [28]. Amplification of the *URA5* gene was performed in a final volume of 50 μL. Each reaction contained 50 ng of DNA, 1X PCR buffer (200 mM Tris-HCl (pH 8.4), 500 mM KCl—Invitrogen), 0.2 mM each of dATP, dCTP, dGTP, and dTTP (Invitrogen), 2 mM magnesium cloride, 1.5 U Taq DNA polymerase (Invitrogen), and 50 ng of each primer URA5 (5’ ATGTCCTCCCAAGCCCTCGACTCCG 3’) and SJ01 (5’ TTAAGACCTCTGAACACCGTACTC 3’). PCR was performed for 35 cycles in a Eppendorf gradient mastercycler (Hamburg, Germany), using the following cycling conditions: 94°C for 2 min initial denaturation, 45 s of denaturation at 94°C, 1 min annealing at 61°C, and 2 min extension at 72°C, followed by a final extension cycle for 10 min at 72°C. PCR products were double digested with *Sau96*I (10 U/μL) and *Hha*I (20 U/μl) for 3 h, and the DNA fragments were separated via 3% agarose gel electrophoresis at 100 V. *URA5*-RFLP patterns were assigned visually by comparing them with the patterns obtained from the standard strains WM 148 (VNI/AFLP1), WM 626 (VNII/AFLP1A), WM 628 (VNIII/AFLP2), WM 629 (VNIV/AFLP3), WM 179 (VGI/AFLP4), WM 178 (VGII/AFLP6), WM 175 (VGIII/AFLP5), and WM 779 (VGIV/AFLP7).

### Mating Type identification

The mating type was identified by PCR using specific primers for the pheromone genes: MFalfaU (5’ TTCACTGCCATCTTCACCACC 3’); MFalfaL (5’ TCTAGGCGATGACACAAAGGG 3’) for mating type **alpha** (MATalpha) and JOHE9787 (5’ ACACCGCCTGTTACAATGGAC 3’); JOHE9788 (5’ CAGCGTTTGAAGATGGACTTT 3’) for mating type **a** (MAT**a**) [3]. Amplification of both genes was performed independently in a final volume of 50μL containing 50 ng of DNA, 1X PCR buffer [200 mM Tris-HCl (pH 8.4), 500 mM KCl—Invitrogen], 0.2 mM each of dATP, dCTP, dGTP, and dTTP (Invitrogen), 2 mM magnesium cloride, 2.5 U Taq DNA polymerase (Invitrogen), and 50 ng of each primer, in a Eppendorf gradient mastercycler (Hamburg, Germany), using the following cycling conditions: 95°C for 3-min initial denaturation, 30 cycles at 94°C for 1 min, annealing at 57.5°C for 1 min, extension at 72°C for 1 min, and a final extension at 72°C for 7 min. The unique fragment corresponding to each mating type was visualized after 3% agarose gel electrophoresis at 100 V.

### MultiLocus Sequence Typing (MLST)

Sub-typing and molecular polymorphism analysis were performed according to the ISHAM consensus multi-locus sequence typing scheme for *C*. *neoformans* and *C*. *gattii* [8] including seven unlinked genetic loci, the genes: *CAP59*, *GPD1*, *LAC1*, *PLB1*, *SOD1*, *URA5* and the IGS1 region. All 7 loci were amplified as previously described [8]. The sequences were manually edited using the software Sequencher 4.10.1 (Gene Codes Corporation, MI, USA), and the allele types (AT) and the combined sequence types (ST) were identified via the MLST webpage http://mlst.mycologylab.org/. The corrected sequences were aligned using the program MEGA version 5 [29]. An un-rooted Neighbor-Joining tree was constructed from the combined loci of all environmental isolates studied using the program MEGA version 5. The genetic distance between isolates was computed using the p-distance, and all positions containing alignment gaps were eliminated in the pairwise sequence comparisons. Bootstrap analysis using 1000 replicates was used to estimate support for clades of the concatenated dataset. The haplotype diversity (Hd) was calculated using DnaSP v5.10 (http://www.ub.edu/dnasp/). Novel genotypes have been submitted to *C*. *gattii* MLST database (http://mlst.mycologylab.org/).

## Results

Fifty one dust samples were obtained from randomly chosen houses in 9 neighborhoods of the city Santa Isabel do Rio Negro, of which three were positive for *C*. *gattii* (Fig. 1, Table 1). The sample SI-443 accounted for 2.500 CFU/g of dark brown colonies, SI-444 for 50 CFU/g and DW-650 exhibited uncountable number of colonies, with an estimated concentration of >50.000 CFU/g. A total of 144 dark brown colonies from the 3 positive houses grown on bird-seed agar were isolated and identified as *C*. *gattii* molecular type VGII. From those, 45 isolates were randomly selected for further characterization of the mating type and MLST analysis, including the single colony from sample SI-444, 14 colonies from sample SI-443, and 30 colonies from sample DW-650. Mating type analysis demonstrated 44 isolates were MATalpha, and one isolate from sample SI-443 was identified as MAT**a** (Table 1).

**Table 1:** List of isolates used in the study from the positive house samples according to the phenotypic identification (NSA, Urea and CGB) and molecular characterization (*URA5*-RFLP type, Mating type and MLST type).

*Positive house*	*Strain*	*NSA*	*Urea*	*CGB*	*URA5-RFLP*	*Mating Type*	*MLST Type*
DW650	CFP352	+	+	+	VGIIb	alpha	ST7
	CFP353	+	+	+	VGII	alpha	ST5
	CFP354	+	+	+	VGII	alpha	ST264
	CFP355	+	+	+	VGII	alpha	ST264
	CFP356	+	+	+	VGIIb	alpha	ST7
	CFP380	+	+	+	VGII	alpha	ST264
	CFP381	+	+	+	VGIIb	alpha	ST7
	CFP382	+	+	+	VGIIb	alpha	ST7
	CFP383	+	+	+	VGIIb	alpha	ST7
	CFP384	+	+	+	VGIIb	alpha	ST7
	CFP385	+	+	+	VGIIb	alpha	ST7
	CFP387	+	+	+	VGIIb	alpha	ST7
	CFP388	+	+	+	VGII	alpha	ST265
	CFP389	+	+	+	VGIIb	alpha	ST7
	CFP390	+	+	+	VGIIb	alpha	ST7
	CFP391	+	+	+	VGIIb	alpha	ST7
	CFP392	+	+	+	VGIIb	alpha	ST7
	CFP393	+	+	+	VGIIb	alpha	ST7
	CFP394	+	+	+	VGIIb	alpha	ST7
	CFP395	+	+	+	VGII	alpha	ST264
	CFP396	+	+	+	VGIIb	alpha	ST7
	CFP397	+	+	+	VGII	alpha	ST5
	CFP398	+	+	+	VGIIb	alpha	ST7
	CFP399	+	+	+	VGIIb	alpha	ST7
	CFP400	+	+	+	VGIIb	alpha	ST7
	CFP401	+	+	+	VGIIb	alpha	ST7
	CFP402	+	+	+	VGIIb	alpha	ST7
	CFP403	+	+	+	VGII	alpha	ST264
	CFP404	+	+	+	VGII	alpha	ST264
	CFP405	+	+	+	VGIIb	alpha	ST7
SI443	CFP409	+	+	+	VGII	alpha	ST266
	CFP406	+	+	+	VGIIa	alpha	ST20
	CFP407	+	+	+	VGIIa	alpha	ST20
	CFP408	+	+	+	VGIIa	alpha	ST20
	CFP410	+	+	+	VGII	a	ST268
	CFP411	+	+	+	VGIIa	alpha	ST20
	CFP412	+	+	+	VGIIa	alpha	ST20
	CFP413	+	+	+	VGIIa	alpha	ST20
	CFP414	+	+	+	VGIIa	alpha	ST20
	CFP415	+	+	+	VGIIa	alpha	ST20
	CFP416	+	+	+	VGIIa	alpha	ST20
	CFP417	+	+	+	VGII	alpha	ST267
	CFP418	+	+	+	VGIIa	alpha	ST20
	CFP419	+	+	+	VGIIa	alpha	ST20
S444	CFP420	+	+	+	VGIIb	alpha	ST7

**Fig 2:**
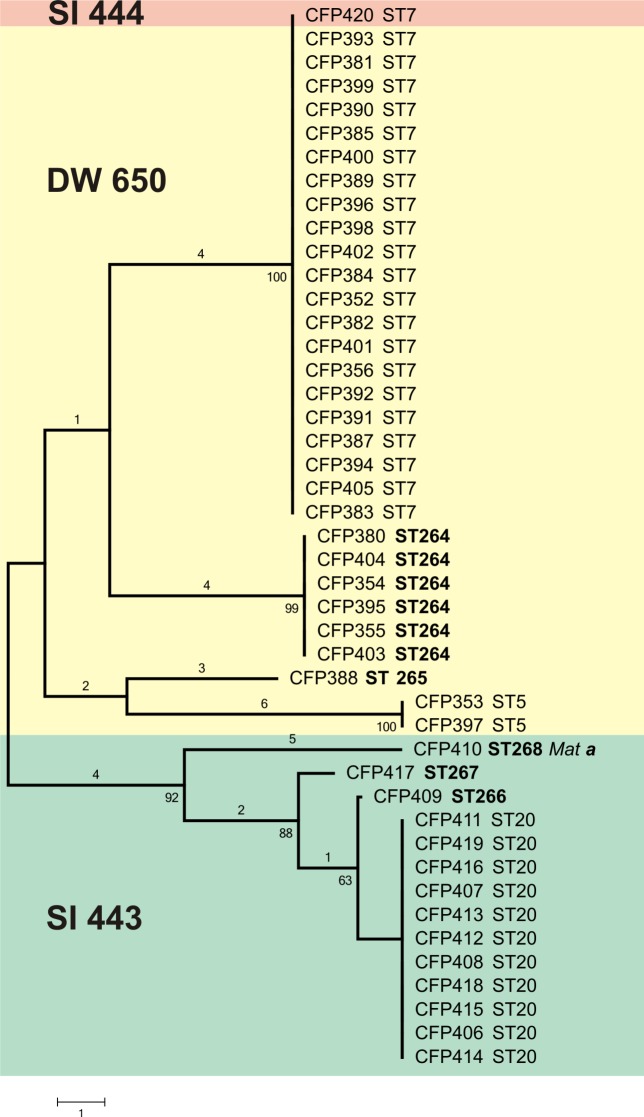
Unrooted neighbor-joining tree inferred from the combined MLST sequences of *CAP59*, *GPD1*, *LAC1*, *SOD1*, *URA5*, *PLB1* genes and the IGS1 region of the 45 strains investigated in this study obtained with the program MEGA version 5. Numbers above the branches are nucleotides differences and below the branches are bootstrap values obtained from 1,000 pseudoreplicates. Sequence types in bold are unique to Brazil.

MLST analysis revealed a total of 8 sequence types among the 45 VGII environmental isolates (Fig. 2, Table 1), and the DNA polymorphism analysis showed extensive haplotype diversity (*H*_*d*_ = 0.695). The most common sequence type was ST7 (VGIIb), representing 48.9% of the isolates (n = 22) analyzed. The second most common was ST20 (VGIIa), representing 24.5% of the studied isolates (n = 11). The ST264 is exclusive for Brazil and represented 13.3% of the isolates (n = 6). ST5 was identified in 2 isolates. The sequence types ST265, ST266, ST267 and ST268 were unique to this region and to Brazil and represented by one isolate each (2.2%). The MLST data have been deposited in the International MLST database for *C*. *neoformans* and *C*. *gattii*, and the corresponding sequences can be obtained in the webpage http://mlst.mycologylab.org/.

## Discussion

Publications on the presence of agents of cryptococcosis in indoor dust are scarce. Two studies, originated from index-cases of aids-associated cryptococcosis in South America and Africa, showed similar results: in Rio de Janeiro, southeast of Brazil, the investigation of 77 dwellings revealed the isolation of *C*. *neoformans* from domestic dust in 11 (14.3%) [21], and some years earlier in Bujumbura, Burundi, *C*. *neoformans* was isolated from domestic dust in 13 (54%) out of 24 dwellings studied [30]. The principal factor associated with domiciliary contamination by *C*. *neoformans* in these studies was the presence of avians in the domestic environment or nearby the homes. The present study has no index-case and revealed the exclusive occurrence of *C*. *gattii* VGII in indoor dust obtained from dwellings in a remote human settlement formed in the heart of the Amazon rainforest in Brazil. A significant positivity, of 5.9%, in indoor dust samples was observed, thus suggesting daily human exposure to these potential disease agents inside those houses. On the contrary to previous reports, no domestic or caged birds were observed in the present study. The houses in Santa Isabel do Rio Negro are rustic, most of them built with planks of wood obtained from the extraction of native species of trees in the surrounding rainforest, especially the Brazil nut tree.

There is much evidence that *C*. *gattii* VGII is widely distributed in Amazonia, e.g. this genotype has been isolated from several species of trees from Brazil and Colombia [31]. Previous findings in anthropic ecosystems in Brazil suggest that members of the *Cryptococcus neoformans/C*. *gattii* species complex are not associated with a particular tree, but with a specialized niche resulting from the natural biodegradation of wood [32]. Moreover, the isolation of *C*. *gattii*, strain LMM645, by Fortes *et al*. 2001 from a native jungle tree (*Guettarda acreana*) on a wild island of the Amazon rainforest, suggests that wild tropical forests may harbour primary sources of this agent. This was recently again emphasised by a global study pointing to the same environmental isolate, LMM645, suggesting an ancient dispersal of the human fungal pathogen *C*. *gattii* from the Amazon rainforest [33]. Lazéra *et al*. in 1996 suggested already that hollows of living trees may provide environments that are sheltered, damp and probably less exposed to changes in climatic conditions, offering more suitable conditions for the survival, adaptation and reproduction of *C*. *gattii* [23]. Likely, the wooden houses in Santa Isabel do Rio Negro also may provide comparable conditions for cryptococcal propagules coming from sources of *C*. *gattii* VGII in the native rainforest, thus forming a microfocus in human dwellings.

Studies on the population structure of the *Cryptococcus neoformans/C*. *gattii* species complex can help the understanding of the geographic expansion and the reproductive strategies of VGII adapted to human dwellings in the Amazon rainforest. The present study revealed an unexpected high genetic diversity amongst the obtained VGII isolates, with eight MLST sequence types (ST20 (= VGIIa), ST7 (= VGIIb), ST5, ST264, ST265, ST266, ST267, and ST268) being present amongst 45 environmental strains from three houses, compared with Australia where only 6 MLST sequence types (ST20 (VGIIa), ST7 (VGIIb), ST5, ST21, ST33, ST38, and ST48) were found amongst 54 clinical, veterinary and environmental isolates from the entire Australian continent [34], and in the USA, where only 7 MLST sequence types, including VGIIa (ST20), VGIIb (ST7) and VGIIc (ST6) were present amongst 212 clinical and veterinary isolates, with most of them originating from the Pacific Northwest [7]. Both studies revealed the presence of a predominant clonal structure amongst most of the VGII populations in Australia and the USA.

The haplotype diversity has till now only been described for *C*. *neoformans*, where it ranged from 0.20 in Asia, 0.40 in South America, 0.46 in Italy, 0.75 in North America and 0.79 in Africa [35]. Concerning *C*. *gattii*, no previous similar analysis has been reported. The VGII haplotype diversity index observed in the present study (HD = 0.695) is impressive, and was based on the analysis of all the seven studied MLST loci, amongst all 45 investigated strains, suggesting a high genetic diversity, especially when considering the very restricted area studied (4 km^2^). The major genotype found in Santa Isabel do Rio Negro, ST7 (= VGIIb), is also the most common one found around the world especially in Australia, Thailand, and caused the minority of the Vancouver Island outbreak in British Columbia, Canada and the Pacific Northwest of the USA (PNW) [4–6, 33,34]. The sequence type ST5, first identified in Australia, was also found in one dwelling (DW650) closely related to ST7 (= VGIIb) and two other new subtypes (ST264, ST265).

In the positive house SI443, approximately 1 km away from the house DW650, the occurrence and distribution of the identified subtypes was totally different. Here the sequence type ST20 (= VGIIa) was predominant, which corresponds to the main agent of the Vancouver Island and Pacific Northwest outbreaks [4,6]. In the same house this sequence type was associated to three more new subtypes (ST266, ST267, ST268). On the other hand, the house SI444, located very close to the house SI443, showed only a single sequence type, ST7 (= VGIIb), which is the same as the one found in the house DW650, located 1km away. The different distribution of subtypes amongst the houses cannot be explained only by mechanical dispersal factors, such as air flow, small animals, wood debris or by shoes. Probably the contamination of the houses by cryptococcal propagules was a past event and most likely resulted from the forest wood of which the houses were constructed. This initiated the process of adaptation of selected strains inside the houses, where they undergo reproduction. The predominance of sequence type ST7 (= VGIIb) in one of the houses, with more than 50.000 CFUs, and only MATalpha suggests an overwhelming clonal, mitotic reproduction mode. On the other hand, the house with the lower density of cryptococcal propagules (2.500 CFU/g), in which both mating types, MATalpha and MATa, were identified, suggest a possible event of alpha-a recombination in nature. Further analysis of dwellings in other areas of the Amazon region, as well as in the same area should allow for a better understanding of the dynamics of the colonization and possible changes in the population structure of *C*. *gattii* VGII.

Our results emphasise that indoor exposure risks to the subtypes of VGII must be considered as a possible infection source, although in the current study no evidence of a disease outbreak, based on the data available, has been observed amongst the residents of Santa Isabel do Rio Negro. Suboptimal diagnosis of cryptococcosis and the absence of surveillance may contribute to the underreporting of cryptococcosis. On the other hand, the frequent exposure to *C*. *gattii* propagules may lead to subclinical infection with regressive pulmonary lesions and acquired natural immunity to cryptococcal infection, warranting a detailed clinical surveillance in the region. Evidence for a direct impact of continuous exposure to infectious propagules from VGII strains in domestic environment is given by the previous reports of a high proportion of cryptococcosis cases due to *C*. *gattii* VGII in the Amazon region of Brazil, in HIV negative children [13, 15].

Over the past two decades, several cryptococcal outbreaks have occurred, including the high-profile ‘Vancouver Island’ and ‘Pacific Northwest’ outbreaks, caused by *C*. *gattii* VGII, which have affected hundreds of otherwise healthy humans and animals [3–7]. To understand the origins of those outbreaks a number of studies have been performed. Fraser *et al*. (2005) initially suggested same-sex mating between to MAT alpha strains involving a low virulent Australian strain (genotype ST7 (= VGIIb) leading to the raise of the high virulent ST20 (= VGIIa) genotype strains and the expansion of the ecological niche of this species. Later studies showed a high genetic diversity among strains from the Amazon rainforest and the possible occurrence of mating between strains of opposite mating types as the source of a global dispersal of this species [33].

In conclusion, the herein described results provide evidence for the later perspective and showed clearly the presence of two different ways of propagation, clonal and recombination, which can lead to the establishment of environmental populations present in indoor dust in urban houses within remote settlements in the Amazon rainforest. These yeast populations are most likely associated with the original sources of the building materials obtained from the surrounding forests. The current study established the basis for future studies investigating the impact of early and continuous exposure to the development of infections and the prevalence of subclinical and clinical infections amongst the inhabitants of houses colonized by *C*. *gattii* VGII.
